# Parallel array with axially coded light-sheet microscope

**DOI:** 10.1038/s41377-020-0310-3

**Published:** 2020-04-20

**Authors:** Pablo Loza-Alvarez

**Affiliations:** ICFO-The Institute of Photonic Sciences, The Barcelona Institute of Science and Technology, 08860 Castelldefels, Barcelona Spain

**Keywords:** Light-sheet microscopy, Imaging and sensing

## Abstract

A parallel array of frequency modulated light sheets results in a scanning-less light sheet microscope capable of fast volumetric imaging.

Light-sheet fluorescence microscopy (LSFM) is a powerful technique to visualize, with high spatial resolution, the dynamics occurring within a volume of a living sample. Many implementations have been presented and optimized to capture faster and faster events occurring in the full 3D volume of the sample. Most of them are normally based on the use of complex setups and synchronization schemes. These unavoidably rely on fast scanning of the light sheet using galvanometric scanning mirrors^1,2^, acousto-optic deflectors^3^, or polygonal rotating mirrors^4^. Then, to properly image the signal generated by the moving light sheet, advanced instrumentation is normally required. These include the use of electrically tunable^5^ or tunable acoustic gradient^3^ lenses, the use of deformable mirrors for wavefront-coding strategies^6^, or even the use of photomultiplier tube arrays^7^ to keep up with the fast image acquisition rates.

On the other hand, there already exist strategies for imaging full volumes of a sample that need no scanning. These methods rely on decoding the information generated from the sample using Fourier domain approaches^8^, compressive sensing^9^, or deconvolution algorithms^10^ based on the frequency or spatial modulation of the excitation beam. These volume illumination approaches can be later demodulated for 3D image reconstruction of the sample. Those schemes do not rely on any light scanning, as the full volume to be imaged is illuminated at once. Such approaches open the possibility to reduce the number of acquisitions for 3D imaging and consequently the acquisition time and the phototoxic effects induced in the sample.

More recently, Ren et al.^11^ introduced a hybrid yet simple setup that takes advantage of illuminating the full sample and, at the same time, uses the potential of LSFM without the need to perform any scanning. This concept was named coded light-sheet array microscopy (CLAM). The key element in CLAM is the use of two static and nearly parallel highly reflective mirrors^12^ that, after a number of reflections, reflect back the incoming beam. This element converts the incident beam into a number of virtual sources that are used to generate a parallel array of light sheets, which are sent into the sample. Then, by using a fast rotating amplitude encoder, each of these light sheets is modulated with a different frequency. The Fourier transform of the signal, captured by each pixel of the camera (in the detection arm of the light-sheet microscope), will then contain frequency components that correspond to a different light sheet and therefore to a different depth in the sample.

In CLAM, by controlling the angle between the two tilted mirrors, the light-sheet array can be reconfigurable in both the number of planes or the array density and coherency. Furthermore, a 100% spatial duty cycle in detection can be achieved, which is translated into a longer voxel dwell time and therefore offers the possibility to increase the signal-to-noise ratio and, therefore, enables a reduced light dose. Finally, this approach can be adapted to any existing LSFM system with minimal hardware or software modification.

The future challenge for CLAM relies on its own nature, as it is based on its capacity to image a number of consecutive parallel light sheets. These require the need for strategies to extend the depth of field, which intrinsically trades-off resolution. Nevertheless, CLAM is a general and versatile technique that can be combined with the multiple strategies normally implemented in LSFM. For example, it can be used in a simultaneous multiview configuration, or can be combined with the use of adaptive optics for aberration correction or, more generally, with wavefront coding/shaping techniques. The additional degree of freedom that it provides allows for the creation of sophisticated patterns, for example, for structured illumination, opening up new possibilities, and applications.
